# MAOA haplotypes associated with thrombocyte-MAO activity

**DOI:** 10.1186/1471-2156-6-46

**Published:** 2005-09-20

**Authors:** Mårten Jansson, Shane McCarthy, Patrick F Sullivan, Paul Dickman, Björn Andersson, Lars Oreland, Martin Schalling, Nancy L Pedersen

**Affiliations:** 1 Department of Medical Epidemiology and Biostatistics, Karolinska Institutet, Stockholm, Sweden; 2 Center for Genomics and Bioinformatics, Karolinska Institutet, Stockholm, Sweden; 3 Departments of Genetics, Psychiatry & Epidemiology, University of North Carolina at Chapel Hill, NC, USA; 4 Department of Neuroscience, Uppsala University, Uppsala, Sweden; 5 Department of Molecular Medicine, Karolinska Institutet, Stockholm, Sweden; 6 Department of Psychology, University of Southern California, Los Angeles, USA

## Abstract

**Background:**

The aim was to ascertain whether thrombocyte MAO (trbc-MAO) activity and depressed state are genetically associated with the MAO locus on chromosome X (Xp11.3 – 11.4). We performed novel sequencing of the MAO locus and validated genetic variants found in public databases prior to constructing haplotypes of the MAO locus in a Swedish sample (N = 573 individuals).

**Results:**

Our results reveal a profound SNP desert in the *MAOB *gene. Both the *MAOA *and *MAOB *genes segregate as two distinct LD blocks. We found a significant association between two *MAOA *gene haplotypes and reduced trbc-MAO activity, but no association with depressed state.

**Conclusion:**

The MAO locus seems to have an effect on trbc-MAO activity in the study population. The findings suggest incomplete X-chromosome inactivation at this locus. It is plausible that a gene-dosage effect can provide some insight into the greater prevalence of depressed state in females than males.

## Background

Monoamine oxidase A (*MAOA*) and B (*MAOB*) are enzymes that deaminate monoamines such as serotonin, dopamine and noradrenaline. The genes encoding *MAOA *and *B *are located on the X chromosome in a tail-to-tail orientation and separated by approximately 20 kilobases (kb) [1,2]. Although *MAOA *and *MAOB *span 65 kb and 116 kb, respectively, both genes display a high degree of homology and most certainly have a common ancestry [3]. The frequencies of confirmed polymorphisms in the two genes vary widely among different ethnic groups [4-6]. Only two common haplotype variants of the *MAOA *locus were found among individuals of northern European ancestry [5].

Both enzymes are localized in the outer mitochondrial membrane [7]. They are also present in glial cells [8], although *MAOA *is less expressed than *MAOB *[9]. The enzymes differ in their expression patterns not only peripherally in the body but also in the central nervous system (CNS) [10]. *MAOB *is the only form that is expressed in human blood cells. *MAOA *is primarily expressed in catecholaminergic neurons in the human brain [10,11], whereas *MAOB *is expressed in serotonergic [10] and histaminergic neurons [8]. The two MAO-enzymes also differ on substrate preferences; *MAOA *preferentially metabolizes serotonin and norepinephrine while *MAOB *has a much higher affinity for phenylethylamine [12,13] and benzylamine [14].

Thrombocyte-MAO activity (Trbc-MAO) has been associated with cerebrospinal fluid (CSF) levels of serotonin metabolites in humans [15] and is higher in women than men [16-18]. This difference has been speculated to be an effect of sex steroids altering the enzyme's activity or a matter of escaped X-inactivation [19]. The proportion of variance in trbc-MAO activity explained by genetic factors (its heritability) in a Swedish population is 77% [20]. Trbc-MAO activity is weakly associated with a C/T polymorphism in intron 13 of the *MAOB *gene in a Swedish population [21] and is also influenced by smoking and specific medications; smokers can have a 30–40% lower trbc-MAO activity than non-smokers [22]. Trbc-MAO activity is also associated with several psychiatric syndromes [23], personality traits and mood disorders e.g. [24-28].

In the present study we address issues concerning genetic variation in *MAOA *and *MAOB *genes, activity levels of trbc-MAO, and associations with depressed state. Genetic variation was analyzed by sequencing the regulatory region of both *MAOA *and *MAOB*, and validating SNPs reported in public databases. We used multiple SNPs covering the MAO gene locus to generate haplotypes on a population level. Finally, we investigated associations between depressed state and trbc-MAO activity and genetic variants in the MAO locus in a large elderly Swedish population.

## Results

### Trbc-MAO activity and depressed state

We found a clearly significant difference between males (mean; 10.7) and females (mean; 12.1) (t = 4.69; p ≤ 0,0001), as well as between smokers and non-smokers in mean trbc-MAO activity (t = 5,86; p =< 0,0001). Smokers showed a 23% lower trbc-MAO activity compared to non-smokers. Females with a depressed state showed a significantly higher mean trbc-MAO activity than unaffected females (t = 2,02; p = 0,04).

### Genetic variants and haplotype construction

Approximately 4.5 kb of both the *MAOA *and *MAOB *gene promotor, including the first exons, totaling 9 kb, were sequenced from a total of 148 X chromosomes. Power to discover SNPs with frequencies greater than 1% and 3% for this sample size was 77% and 100%, respectively. No variants were found in the *MAOB *gene. In contrast, one previously reported variation was confirmed (rs3788863) for the *MAOA *gene, lying within the first intron, as well as two additional variants further down stream with a minor allele frequency greater than 1%. Both the recorded and most distal variants showed complete LD with each other, therefore only the recorded variant was chosen for further analysis.

The genotyping error rate was calculated at 0,4% through males scoring as heterozygotes and from MZ twins where both twins in a pair were genotyped. These errors could not be scored differently from the sequence and therefore most likely reside in the handling of samples, e.g. contamination or labelling error.

In addition to resequencing the upstream regions, we genotyped reported SNPs in the remainder of the gene clusters by Pyrosequencing. Six of the previously reported SNPs could not be confirmed as polymorphic (rs1014876, rs3027464, rs6324, rs1040398 and two SNPs reported by Balciuniene et al.) The remaining nine polymorphic variants; one in the Norrie gene (rs766117), four SNPs in *MAOB *(rs1181252, rs2283729, rs3027452 and rs1799836) and four SNPs in *MAOA *(rs1801291, rs979605, rs6323, rs388863) were sequenced in the total sample.

**Figure 2:**
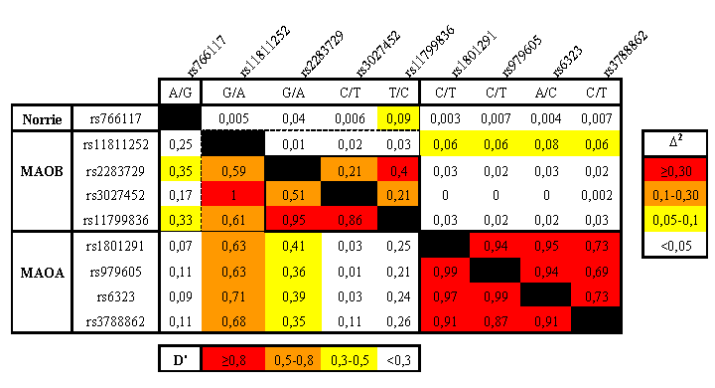
**LD map**. Pair-wise LD map with one individual from each female pair (N = 356). D' is shown below the diagonal and Δ^2 ^above the diagonal. Color code D': Red: ≥0,8 Orange: 0,5–0,8 Yellow: 0,3–0,5 White: <0,3. Color code Δ^2^: Red: ≥0,30 Orange: 0,1–0,30 Yellow: 0,05–0,1 White: <0,05.

The LD map (Figure 2) displays a clear structure of the MAO locus with strong LD across the *MAOA *gene in a distinct block spanning approximately 65 kb. The *MAOB *gene also displays a similar block-like structure, although the pattern of LD is not as robust as for *MAOA*. This is perhaps due to the inconsistencies in allele frequencies across *MAOB*. Interestingly, weak LD is observed at the tail ends between the two MAO loci.

Furthermore, because there was no LD between the Norrie gene variant, located >66 kb upstream of *MAOB*, and any other variant in the MAO region, we decided not use this variant further in the haplotype assessment. Modest deviations from Hardy-Weinberg equilibrium were noted in rs766117 in the Norrie gene (p = 0,022) and rs979605 in intron 10 of the *MAOA *gene (p = 0,028). This could reflect the underlying LD structure [29], as demographic influences would act over larger regions [30]. However to clarify this, a denser set of SNPs would need to be genotyped.

**Figure 1:**
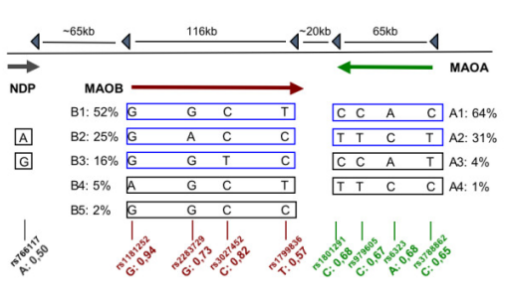
**Genetic structure of the MAO locus**. Haplotype and common allele frequencies in the total sample. dbSNP rs numbers for all genotyped SNPs are presented with major allele frequencies. Haplotypes frequencies illustrated for *MAOA *and *B *separately as well as the genes combined (See Text). NDP was not used in the haplotype frequency estimations.

In the male population we could identify five distinct haplotypes in the *MAOB *gene and four in the *MAOA *gene with frequencies ≥1% (Figure 1.). When analyzing the MAO locus as one large block using eight SNPs, we found ten distinct locus haplotypes with a frequencies ≥1% (data not shown). In the female population, "PHASE" assembled identical higher frequency haplotypes as were identified in the male sample, with minor discrepancies in lower frequency haplotypes due to unknown phase (Figure 1).

### Associations with SNPs

In the total sample, no single variant of any of the individual SNPs was associated with trbc-MAO activity. However, in females the C/C and C/T genotypes of rs979605 in the *MAOA *gene were associated with a significant decrease in trbc-MAO activity, (-2,9; CI 95%: -5,2 – -0,6) and (-2,4; CI 95%: -4,7 – -0,1) respectively.

Analyzed by gender, depressed state was associated with the A-allele of *MAOB *SNP rs1181252 in males (OR = 4,5; CI 95%: 1,0 – 21,7) and both GG and GA of rs766117 (OR = 2,2; CI 95%: 1,1 – 4,3) in females. It should be noted that the "A" allele of rs1181252 only had a population frequency of 6%.

### Associations with haplotypes

There was no association between any of the *MAOB *haplotypes and trbc-MAO activity. Two *MAOA *haplotypes, A1 and A3, both sharing identical alleles at the three first haplotype positions (CCA-) (Figure 1), were associated with a significant decrease in trbc-MAO activity (Table 1). Analyses of the entire MAO locus and trbc-MAO activity did not reveal any significant findings (data not shown). We could not find any significant associations between depressed state and any specific haplotype in men or women (Table 2).

**Table 1:** Associations between MAO haplotypes and trbc-MAO activity, reported as unit change in mean trbc-MAO activity per allele and controlling for gender and smoking status.

	Total sample N = 340	Males N = 156	Females N = 184
	per allele	Hemizygous	per allele

Haplotypes	Estimates (Unit change in mean trbc-MAO activity per allele)		
B1	-0,38 (-1,3 – 0,5)	0 (ref)	-0,3 (-1,1 – 0,5)
B2	-0,63 (-1,8 – 0,5)	-0,08 (-1,6 – 1,5)	-0,4 (-1,5 – 0,7)
B3	-0,18 (-1,6 – 1,3)	-0,8 (-2,9 – 1,2)	-0,2 (-1,5 – 1,0)
B4	-1,3 (-3,5 – 0,8)	0,7 (-2,8 – 4,3)	-1,0 (-2,8 – 0,8)
B5	-1,7 (-5,1 – 1,6)	NA	-0,7 (-3,8 – 2,4)
Male gender	-2,1 (-3,3 – -0,9)*		
Non-smokers	2,3 (1,1 – 3,5)*	1,5 (0,2 – 2,8)	3,5 (2,0 – 5,0)

A1	-1,1 (-1,9 – -0,3)*	-1,8 (-3,2 – -0,5)*	-1,0 (-1,7 – -0,3)*
A2	0,1 (-0,9 – 1,2)	0 (ref)	0,6 (-0,4 – 1,5)
A3	-3,1 (-6,1 – -0,14)*	-2,3 (-5,8 – 1,2)	-4,1 (-7,4 – -0,7)*
A4	-0,02 (-3,8 – 3,7)	NA	-0,5 (-3,7 – 2,7)
Male gender	-2,3 (-3,5 – -1,1)*		
Non-smokers	2,4 (1,1 – 3,6)*	1,4 (0,03 – 2,8)	3,4 (1,9 – 4,8)

**Table 2:** MAO haplotypes and depressive state, reported as odds ratios per allele. Without genetic information in the model male gender was significant [OR: 0,5 (0,3 – 0,8)] for depressive state. *Homozygote compared to heterozygote.

MAO haplotypes and depressive state				
	Total sample N = 573	Males N = 239	Females N = 334	

	per allele	Hemizygous	per allele	Homozygotes*

	Odds Ratio with 95% CI			
B1	1,2 (0,6 – 2,5)	1,0 (ref)	1,4 (0,6 – 2,9)	1,3 p = 0,57
B2	1,5 (0,7 – 3,3)	1,7 (0,8 – 3,6)	1,5 (0,6 – 3,3)	1,2 p = 0,80
B3	1,3 (0,5 – 3,0)	0,7 (0,3 – 1,7)	1,7 (0,7 – 4,2)	1,9 p = 0,51
B4	2,0 (0,8 – 5,2)	3,7 (0,7 – 18,7)	2,0 (0,7 – 5,3)	1,7 p = 0,59
B5	0,5 (0,1 – 2,8)	2,4 (0,4 – 14,5)	0,3 (0,04 – 2,5)	NA
Male gender	0,7 (0,3 – 1,5)			

A1	3,0 (0,8 – 12,2)	1,0 (ref)	2,2 (0,6 – 8,4)	5,5 p = 0,08
A2	2,5 (0,6 – 10,6)	0,9 (0,4 – 1,8)	1,7 (0,4 – 7,1)	1,3 p = 0,80
A3	2,8 (0,7 – 11,4)	1,2 (0,3 – 4,4)	1,7 (0,4 – 6,9)	NA
A4	3,2 (0,6 – 18,6)	NA	2,8 (0,4 – 17,3)	NA
Male gender	1,4 (0,3 – 6,0)			

When the model was analyzed without genetic information, males have a significantly lower risk for being affected with depressed state compared to women (OR = 0,5). This gender effect may be explained by the genetic information (even though no associations were found with any of the haplotypes), because the risk for depressed state due to the male gender is differs in the analyses of the *MAOA *locus (OR = 1,4; non-significant) and the *MAOB *locus, where the estimate is similar to the model without genetic information.

Interestingly, in females all *MAOB *homozygote haplotypes displayed greater odds ratios with depressed state than that for heterozygotes (Table 2), indicating an additive effect. The same was true for *MAOA *(Table 2).

## Discussion

Monoamine oxidase A and B constitute two important molecules in the human body in general and in the central nervous system (CNS) in particular. Numerous studies suggest a contribution of these two mitochondrial enzymes to complex human behaviors [26-28,31-33]. In the present study we searched the MAO locus for novel genetic variants and evaluated the genetic and haplotype structure in a Swedish population. We also assessed associations between trbc-MAO activity and depressed state, and their respective associations with the genetic structure of the MAO locus. The key findings of this study are first: the profound lack of variation at functional regions of the two MAO genes and a pattern of two distinct genetic LD blocks, one for each gene. Second: we replicated the gender differences in trbc-MAO activity and demonstrated an association between trbc-MAO activity and depressed state in women. Third: two *MAOA *haplotype variants were associated with decreased trbc-MAO activity although we could not replicate a previously reported genetic association between the *MAOB *gene and trbc-MAO activity. Fourth: we could not find any significant associations between the genetic variants and depressed state. On the other hand, there was an interesting, although not significant dose-response effect of haplotypes displayed in women, with greater odds ratios in homozygotes than heterozygotes.

Considering the size and importance of the MAO locus, relatively few polymorphic sites have been verified. We observed two new variants through sequence screening a partial region of *MAOA *intron 1, but in *MAOB *neither the previously reported nor any novel variants were found in the areas sequenced. It is surprising that so few SNPs were discovered given our power to detect variants with very low frequencies. SNP deserts have been previously noted on the q arm of the X chromosome [34]. Gilad and colleagues [4] have described similar features across *MAOA*, where extensive LD and low nucleotide diversity suggest recent action by population structure forces and perhaps a recent positive selection sweep [35]. Although we could not evaluate the influence of such forces, evidence of strong LD and the lack of decay across *MAOA *in our Swedish sample complement these previous findings. Linkage disequilibrium decays rapidly between the two MAO genes (separated by approximately 20 kb). Perhaps selection is in action much more locally than would be expected in each MAO gene, both separated by regions of higher recombination than that within each gene.

Previous studies have indicated that the *MAOA *gene may harbour relatively few haplotypes within a block structure [5]. We observed similar results here with two haplotypes encompassing 95% of the haplotypic variation. We found similar results for the *MAOB *gene, with a distinct block structure in which three haplotypes explain 93% of the variation. So few haplotypes over such long distances have been observed previously (McCarthy et al, manuscript) and are proposed signatures of selection and population substructure on the X chromosome [36,37].

A previous Swedish association between the *MAOB *gene and trbc-MAO activity [21] could not be replicated nor distinctly refuted, as we found a small non-significant effect of the same allele in males. However, none of the haplotype blocks carrying this allele could strengthen or support this effect, suggesting that this allele is not in high LD with a larger region of the *MAOB *gene.

Two *MAOA *haplotypes (A1 and A3) showed a significant association with reduced trbc-MAO activity. Both haplotypes shared the initial sequence variants [CCA], but varied at the fourth allele [T/C]. Given that only *MAOB *is expressed in platelets there is no clear explanation for this finding. Given the minor kinetic differences between platelet and brain MAO-B [38] and the correlation of *MAOB *and *MAOA *levels in regions of the brain [39], this association may reflect *MAOA *activity in the brain. On the other hand, it is possible that the *MAOA *locus holds cis-acting regulatory elements affecting *MAOB *expression. Another possible explanation could be that one or several single-base variants affected by methylation cause changes in the expression pattern [40].

Our study is based on a relatively large population-based sample of normally aging adults, although it is not without its limitations. We have controlled for smoking, but were unable to do so for intake of certain medications. The study sample was included in a larger study where associations between depressed state and the serotonin receptor 2A and the serotonin transporter were evaluated [41]. The influence of these genes has not been corrected for in the analysis.

## Conclusion

Good et al [19] demonstrated that trbc-MAO activity is related to the number of X chromosomes. We replicated a significant difference in trbc-MAO activity between males and females reported by others e.g. [17]. The findings suggest incomplete X chromosome inactivation at this locus and are consistent with other findings of genes escaping inactivation on the X chromosome [42,43]. It has been hypothesized that this dosage imbalance between males and females might be crucial for gender characteristics [19,44]. Recently it was demonstrated that a number of genes, including *MAOA*, escape X-inactivation [45]. Furthermore, the X-inactivation pattern, which shows a substantial heritability [46], increases in the elderly. Although we could not find a significant association between variants of *MAOB *or *MAOA *and depressed state in this population, we found an interesting dose-response effect in women, with a higher risk for depressed state with homozygosity. Whether levels of trbc-MAO activity are correlated with the number of X chromosomes and whether this might be linked to the higher prevalence of depressive symptoms in females deserves further investigation. Nevertheless it is plausible that a partially doubled gene activity on the X chromosome can explain difference in prevalence of depressive state in men and women.

## Methods

### Participants

The participants were taken from a longitudinal twin study of aging, the Swedish Adoption/Twin Study of Aging (SATSA) with up to five occasions of measurement [47]. SATSA is a sub-sample of the population based Swedish Twin Registry [48]. All participants are Caucasian and born in Sweden. For the present analyses we selected all individuals who participated in an in-person testing session during which questionnaires were administered and a blood sample was drawn. The mean age of the sample was 61,3 years at the time of testing. Twenty two percent of the participants were current smokers; 35% of the males and 15% of the females.

Zygosity was initially based on self-reports of similarity and confirmed by serological analyses and comparisons of up to 10 DNA markers.

For preliminary screening of the promoter, the first exon and intron regions for novel variants, 94 Swedish male blood donors were randomly selected from a larger sample set collected to study *MAOB *regulation. All were between the ages of 20 to 40 years and non-smokers.

This study was reviewed and approved by the Ethics Committee of the Karolinska Institute, the Swedish Data Inspection Board, and the IRBs at the University of Southern California and the Pennsylvania State University. All subjects provided informed consent.

### DNA and trbc-MAO activity

DNA samples were available from 573 twins. Trbc-MAO activity measures were available from 565 twins. The trbc-MAO activity is expressed as nmoles of 2-phenylethylamine oxidized per minute and per 10^10 ^platelets. Trbc-MAO activity measures have previously been described in detail [20].

### Depressed state

Depressive symptoms were measured with the Center for Epidemiologic Studies Depression Scale (CES-D), a 20-item self-report instrument developed for use in the community and well established for use with older adults [49,50]. The scale has been shown to have minimal overlap with physical illness [51] and assesses current symptoms during the past week. Respondents scoring 16 or higher on the CES-D scale are considered to have a clinically relevant depressed state. In this study population of 574 participants, 144 were classified as having a depressed state, 17.9% of the males and 30.2% of females.

### Genotyping & sequencing

Approximately 4.5 kb of each gene was initially sequenced in search of novel SNPs in both *MAOA *and *MAOB*, first in 94 Swedish males and later 45 twins with CES-D scores (36 males and 9 females). Power to detect minor allele frequencies (q) between 1 and 5% was determined as by Glatt et al. [52], 1-(1-q)^N ^where N is the number of chromosomes. Amplification and nested sequencing primers were designed with the CPrimers programme from Genbank entry GI:8671203 containing the promoter, coding exon 1 and flanking intronic sequence of *MAOA *(~5.0 kb, nucleotides 46490–51454) and Genbank entry GI:2440066 spanning the same characterized sequences of *MAOB *(~4 kb nucleotides 35033–39021).

Direct sequencing reactions were performed using DYEnamic ET Dye Terminator Cycle Sequencing Kit (Amersham Biosciences) and separated using a Megabace 1000. Reads were base called with Phred [53], assembled using Phrap and viewed using Consed Version 13 [54]. All SNPs were documented and cross validated with dbSNP at NCBI.

Twelve SNPs identified from public databases (dbSNP at NCBI) and two novel SNPs previously reported (introns 3 and 10 of *MAOB*) a Swedish sample [5] were sequenced in 95 participants (142 chromosomes) by Pyrosequencing to confirm their presence in this population. For Pyrosequencing, either the forward or the reverse primer in each primer pair was biotinylated. Sequencing primers with a length of 14 and 18 bases were placed within one base of the SNP. The PCR reaction was performed in a 50 μl reaction volume, containing 5 ng of genomic DNA, 10 pmoles of each primer, 0.2 mM of each dNTP, 1.5 mM MgCl_2 _and 1.5 U of Taq. Thermal cycling was performed in a PTC-225 DNA machine (MJ Research Inc., Cambridge, MA, USA) at 95°C for 5 min followed by 50 cycles of 95°C for 30 s, 45 s of annealing at an optimized temperature, followed by 72°C for 30 s and a final extension of 5 min at 72°C. The biotinylated PCR product was immobilized onto streptavidin-coated sepharose beads and DNA strands were separated by denaturation with 0.2 M NaOH. The pyrosequencing reaction was performed on a PSQ96™ Instrument from Pyrosequencing AB (Uppsala, Sweden) as described by [55,56]. Detailed primer and assay information are available upon request.

### Statistical analysis

Male haplotypes could be extrapolated directly since the MAO locus is located on the X chromosome and males are thereby hemizygous. Female bi-allelic haplotypes were estimated using an EM algorithm (Sham 1998) and the pair-wise LD measures D' [57] and Δ^2 ^[58]. We used "PHARE" (by David G Cox, available at ) to create input files for "PHASE" [59,60] to construct female haplotypes.

We used linear regression to estimate the association between trbc-MAO activity and genotypic information using a generalized estimating equation (GEE) approach and alternating logistic regression (ALR) [61] to estimate the association between depressed state and genotypic information. We first modeled the association between single SNPs and each of the two outcomes and then modeled the association between haplotype constructs and the two outcomes. All estimates were adjusted for current smoking status. We estimated both dominance and co-dominance models. Explanatory variables in the dominance models were binary whereas in the co-dominance models they were coded as the number of reference alleles (i.e., 0, 1, or 2 for females and 0 or 1 for males). The parameter estimates for the co-dominance models represent the change in the outcome (trbc-MAO activity or odds of being in a depressed state) per affected allele. Due to the continuous nature of the trbc-MAO measure, only one individual from each complete twin pair and single participating individuals were analyzed (N = 340). Among females we also estimated the effect of being homozygote compared to heterozygote. If the co-dominance model is a good fit to the data then these estimates should be similar to the "per allele" estimates from the co-dominance model. All statistical analyses were performed in SAS 8.01 using GENMOD procedure (SAS Institute Inc. Cary, NC).

## Authors' contributions

MJ: Design of the study, performed data analysis and interpretation of data. Carried out the molecular genetic studies (genotyping) and drafted the manuscript.

SM: Participated in the design of the study. Carried out the molecular genetic studies (sequencing), sequence alignment and critically revised the manuscript.

PFS: Participated in the design of the study and critically revised the manuscript for important intellectual content.

PD: Planed and performed the statistical analysis.

BA: Participated in the design of the study and critically revised the manuscript.

LO: Substantially revised the manuscript for important intellectual content.

MS: Participated in the design of the study and critically revised the manuscript.

NLP: Participated in the design of the study and substantially revised the manuscript for important intellectual content.

All authors read and approved the final manuscript.
